# Supplementary material for: Reduced neutralisation of the Delta (B.1.617.2) SARS-CoV-2 variant of concern following vaccination
Source: PLoS Pathog. 2021 Dec 2;17(12):e1010022. doi: 10.1371/journal.ppat.1010022 (PMC8639073; doi:10.1371/journal.ppat.1010022)
Supplement: S1 Table — Mean neutralisation of viral variants by DOVE study sera were grouped by vaccine (BNT162b2 or ChAdOx1) and dose (one or two). Fold reduction was calculated by comparing group means. Fold reduction was also calculated from the data after exclusion of the 8/162 samples that possessed N-reactive antibodies by ELISA (Fold (- N +ves)). Significant differences between groups were calculated using One-way ANOVA and Tukey’s multiple comparisons test (p values) using GraphPad Prism version 8. (DOCX) [file ppat.1010022.s001.docx]

|  | **ChAdOx1** | | **BNT162b2** | |
| --- | --- | --- | --- | --- |
|  | **Dose 1** | **Dose 2** | **Dose 1** | **Dose 2** |
| *n* | 50 | 18 | 38 | 51 |
|  |  |  |  |  |
| Minimum | 25 | 25 | 21 | 22 |
| 25% Percentile | 48.75 | 50.25 | 35 | 34 |
| **Median** | **55.5** | **58**** | **43** | **43**** |
| 75% Percentile | 58.25 | 62.5 | 65 | 50 |
| Maximum | 64 | 87 | 72 | 65 |
| Range | 39 | 62 | 51 | 43 |
|  |  |  |  |  |
| **95% CI of median** |  |  |  |  |
| Actual confidence level | 96.72% | 96.91% | 96.64% | 95.11% |
| Lower confidence limit | 52 | 51 | 38 | 39 |
| Upper confidence limit | 57 | 61 | 54 | 45 |
|  |  |  |  |  |
| **Mean** | **51.36** | **56.56** | **46.11** | **41.47** |
| Std. Deviation | 10.61 | 13.26 | 15.25 | 10.54 |

**S1 Table. Age distribution of DOVE study population.** Median and mean were calculated using GraphPad Prism version 8, descriptive statistics. Age distributions between groups were compared using One-way ANOVA and Tukey’s multiple comparisons test, ** p<0.0001.
