# Supplementary material for: Reduced neutralisation of the Delta (B.1.617.2) SARS-CoV-2 variant of concern following vaccination
Source: PLoS Pathog. 2021 Dec 2;17(12):e1010022. doi: 10.1371/journal.ppat.1010022 (PMC8639073; doi:10.1371/journal.ppat.1010022)
Supplement: S2 Table — Median and mean were calculated using Graphpad Prism, descriptive statistics. Age distributions between groups were compared using One-way ANOVA and Tukey’s multiple comparisons test, ** p<0.0001. (DOCX) [file ppat.1010022.s002.docx]

**S2 Table. Fold reduction in neutralisation by viral variant.** Mean neutralisation of viral variants by DOVE study sera were grouped by vaccine (BNT162b2 or ChAdOx1) and dose (one or two). Fold reduction was calculated by comparing group means. Fold reduction was also calculated from the data after exclusion of the 8/162 samples that possessed N-reactive antibodies by ELISA (Fold (- N +ves)). Significant differences between groups were calculated using One-way ANOVA and Tukey’s multiple comparisons test (p values) using GraphPad Prism version 8.

|  | **All vaccinates** | | | | | | | | | | | |
| --- | --- | --- | --- | --- | --- | --- | --- | --- | --- | --- | --- | --- |
| **Test details** | | **Mean 1** | **Mean 2** | **Mean Diff.** | **SE of diff.** | **n** | **Fold reduction** | **Fold (- N +ves)** | **Significant?** | | **Summary** | **Adjusted P value** |
| **WUHAN vs. B.1.617.1** | | **4562** | **1059** | **3503** | **602.5** | **162** | **4.31** | **4.49** | | **Yes** | ******** | **<0.0001** |
| **WUHAN vs. B.1.617.2** | | **4562** | **893.6** | **3669** | **623.6** | **162** | **5.11** | **6.21** | | **Yes** | ******** | **<0.0001** |
| **WUHAN vs. B.1.351** | | **4562** | **724.9** | **3837** | **578.4** | **162** | **6.29** | **6.06** | | **Yes** | ******** | **<0.0001** |
|  | **BNT162b2 one dose only** | | | | | | | | | | | |
| **Test details** | | **Mean 1** | **Mean 2** | **Mean Diff.** | **SE of diff.** | **n** | **Fold reduction** | **Fold (- N +ves)** | | **Significant?** | **Summary** | **Adjusted P value** |
| **WUHAN vs. B.1.617.1** | | **2500** | **617.6** | **1882** | **610.7** | **37** | **4.05** | **3.40** | | **No** | **ns** | **0.1214** |
| **WUHAN vs. B.1.617.2** | | **2500** | **1777** | **722.6** | **675.9** | **37** | **1.41** | **1.92** | | **No** | **ns** | **0.8233** |
| **WUHAN vs. B.1.351** | | **2500** | **196.3** | **2304** | **531** | **37** | **12.74** | **11.55** | | **Yes** | ******* | **0.023** |
|  | **BNT162b2 two doses only** | | | | | | | | | | | |
| **Test details** | | **Mean 1** | **Mean 2** | **Mean Diff.** | **SE of diff.** | **n** | **Fold reduction** | **Fold (- N +ves)** | | **Significant?** | **Summary** | **Adjusted P value** |
| **WUHAN vs. B.1.617.1** | | **11473** | **1476** | **9997** | **777.7** | **50** | **7.77** | **7.49** | | **Yes** | ******** | **<0.0001** |
| **WUHAN vs. B.1.617.2** | | **11473** | **1015** | **10458** | **775.4** | **50** | **11.30** | **11.14** | | **Yes** | ******** | **<0.0001** |
| **WUHAN vs. B.1.351** | | **11473** | **1200** | **10273** | **753.1** | **50** | **9.56** | **9.30** | | **Yes** | ******** | **<0.0001** |
|  | **ChAdOx1 one dose only** | | | | | | | | | | | |
| **Test details** | | **Mean 1** | **Mean 2** | **Mean Diff.** | **SE of diff.** | **n** | **Fold reduction** | **Fold (- N +ves)** | | **Significant?** | **Summary** | **Adjusted P value** |
| **WUHAN vs. B.1.617.1** | | **637.2** | **444.3** | **192.9** | **349.7** | **50** | **1.43** | **1.43** | | **No** | **ns** | **0.9026** |
| **WUHAN vs. B.1.617.2** | | **637.2** | **159.8** | **477.4** | **264** | **50** | **3.99** | **4.01** | | **No** | **ns** | **0.1841** |
| **WUHAN vs. B.1.351** | | **637.2** | **139.1** | **498.1** | **260.6** | **50** | **4.58** | **4.85** | | **No** | **ns** | **0.1511** |
|  | **ChAdOx1 two doses only** | | | | | | | | | | | |
| **Test details** | | **Mean 1** | **Mean 2** | **Mean Diff.** | **SE of diff.** | **n** | **Fold reduction** | **Fold (- N +ves)** | | **Significant?** | **Summary** | **Adjusted P value** |
| **WUHAN vs. B.1.617.1** | | **1325** | **1914** | **-589.2** | **892.4** | **18** | **0.69** | **1.03** | | **No** | **ns** | **0.9115** |
| **WUHAN vs. B.1.617.2** | | **1325** | **330.1** | **994.9** | **606.8** | **18** | **4.01** | **3.14** | | **No** | **ns** | **0.366** |
| **WUHAN vs. B.1.351** | | **1325** | **897.1** | **427.9** | **693** | **18** | **1.48** | **1.43** | | **No** | **ns** | **0.926** |
