# Supplementary material for: Reduced neutralisation of the Delta (B.1.617.2) SARS-CoV-2 variant of concern following vaccination
Source: PLoS Pathog. 2021 Dec 2;17(12):e1010022. doi: 10.1371/journal.ppat.1010022 (PMC8639073; doi:10.1371/journal.ppat.1010022)
Supplement: S3 Table — Antibody responses measured by pseudotype-based neutralisation assay against Wuhan-hu-1 were compared with those against B.1.617.1, B.1.617.2 and B.1.351. Responses were compared for A) BNT162b2 1 dose, B) BNT162b2 2 doses, C) ChAdOx1 1 dose and D) ChAdOx1 2 doses. Correlations between groups were evaluated and non-parametric Spearman correlation coefficients calculated using GraphPad Prism version 8. (DOCX) [file ppat.1010022.s003.docx]

|  |  | **BNT162b2 1 dose** | | | | | | **BNT162b2 doses** | | | | | | **ChAdOx1 1 dose** | | | | | | **ChAdOx1 2 doses** | | | | | |
| --- | --- | --- | --- | --- | --- | --- | --- | --- | --- | --- | --- | --- | --- | --- | --- | --- | --- | --- | --- | --- | --- | --- | --- | --- | --- |
|  |  | Wuhan  vs. B.1.617.1 | | Wuhan vs. B.1.617.2 | | Wuhan vs. B.1.351 | | Wuhan vs. B.1.617.1 | | Wuhan vs. B.1.617.2 | | Wuhan vs. B.1.351 | | Wuhan vs. B.1.617.1 | | Wuhan vs. B.1.617.2 | | Wuhan vs. B.1.351 | | Wuhan vs. B.1.617.1 | | Wuhan vs. B.1.617.2 | | Wuhan vs. B.1.351 | |
| Spearman r | 0.7093 | | 0.7113 | | 0.3783 | | 0.2555 | | 0.5599 | | 0.6986 | | 0.5077 | | 0.5118 | | 0.4466 | | 0.2138 | | 0.1189 | | 0.6177 | |  |
| 95% confidence interval | 0.49 to 0.84 | | 0.49 to 0.84 | | 0.05 to 0.63 | | -0.03 to 0.50 | | 0.32 to 0.72 | | 0.51 to 0.82 | | 0.25 to 0.69 | | 0.26 to 0.69 | | 0.18 to 0.64 | | -0.29 to 0.62 | | -0.38 to 0.56 | | 0.19 to 0.84 | |  |
| P (two-tailed) | <0.0001 | | <0.0001 | | 0.021 | | 0.0733 | | <0.0001 | | <0.0001 | | 0.0002 | | 0.0001 | | 0.0012 | | 0.3943 | | 0.6384 | | 0.0063 | |  |
| P value summary | **** | | **** | | * | | ns | | **** | | **** | | *** | | *** | | ** | | ns | | ns | | ** | |  |
| Significant? (alpha = 0.05) | Yes | | Yes | | Yes | | No | | Yes | | Yes | | Yes | | Yes | | Yes | | No | | No | | Yes | |  |
| Number of XY Pairs | 37 | | 37 | | 37 | | 50 | | 50 | | 50 | | 50 | | 50 | | 50 | | 18 | | 18 | | 18 | |  |

**S3 Table. Correlation between neutralising antibody titres against vaccine (Wuhan-hu-1) and VOCs**. Antibody responses measured

by pseudotype-based neutralisation assay against Wuhan-hu-1 were compared with those against B.1.617.1, B.1.617.2 and B.1.351.

Responses were compared for A) BNT162b2 1 dose, B) BNT162b2 2 doses, C) ChAdOx1 1 dose and D) ChAdOx1 2 doses. Correlations

between groups were evaluated and non-parametric Spearman correlation coefficients calculated using GraphPad Prism version 8.
