# Supplementary material for: Reduced neutralisation of the Delta (B.1.617.2) SARS-CoV-2 variant of concern following vaccination
Source: PLoS Pathog. 2021 Dec 2;17(12):e1010022. doi: 10.1371/journal.ppat.1010022 (PMC8639073; doi:10.1371/journal.ppat.1010022)
Supplement: S1 Fig — S1 ELISA absorbance 450nm and neutralising antibody titre in the pseudotype-based neutralisation assay against Wuhan-hu-1, B.1.617.1, B.1.617.2 or B.1.351, were compared for each vaccine study group (ChAdOx1 1 dose, ChAdOx1 2 doses, BNT162b2 1 dose and BNT162b2 2 doses). Neutralising antibody titres are expressed as mean (n = 3) +/- SE. (DOCX) [file ppat.1010022.s004.docx]

**S1 Figure. Correlation between antibody responses measured by ELISA and neutralization assay.**
