# Supplementary material for: Reduced neutralisation of the Delta (B.1.617.2) SARS-CoV-2 variant of concern following vaccination
Source: PLoS Pathog. 2021 Dec 2;17(12):e1010022. doi: 10.1371/journal.ppat.1010022 (PMC8639073; doi:10.1371/journal.ppat.1010022)
Supplement: S2 Fig — Antibody responses measured by pseudotype-based neutralisation assay against Wuhan-hu-1, B.1.617.1, B.1.617.2 and B.1.617.2 were stratified into three groups: titre <50; a titre of 50 to 500; and a titre of >500. The percentage of samples in each group were then plotted for the ChAdOx1 1 dose, ChAdOx1 2 doses, BNT162b2 1 dose and BNT162b2 2 doses. (DOCX) [file ppat.1010022.s005.docx]

**S2 Figure. Stratification of vaccine study groups by antibody titre.**
