# Supplementary material for: Reduced neutralisation of the Delta (B.1.617.2) SARS-CoV-2 variant of concern following vaccination
Source: PLoS Pathog. 2021 Dec 2;17(12):e1010022. doi: 10.1371/journal.ppat.1010022 (PMC8639073; doi:10.1371/journal.ppat.1010022)
Supplement: S3 Fig — Antibody responses measured by pseudotype-based neutralisation assay against Wuhan-hu-1 were compared with those against B.1.617.1, B.1.617.2 and B.1.351. Responses were compared for A) BNT162b2 1 dose, B) BNT162b2 2 doses, C) ChAdOx1 1 dose and D) ChAdOx1 2 doses. Correlations between groups were evaluated and non-parametric Spearman correlation coefficients calculated. (DOCX) [file ppat.1010022.s006.docx]

**S3 Figure. Correlation between neutralising antibody titre against Wuhan-hu-1 and variants of concern.**
