# Supplementary material for: Reduced neutralisation of the Delta (B.1.617.2) SARS-CoV-2 variant of concern following vaccination
Source: PLoS Pathog. 2021 Dec 2;17(12):e1010022. doi: 10.1371/journal.ppat.1010022 (PMC8639073; doi:10.1371/journal.ppat.1010022)
Supplement: S4 Fig — Antibody responses measured by pseudotype-based neutralisation assay against Wuhan-hu-1, B.1.617.1, B.1.617.2 and B.1.617.2 were compared with time post-dose 1 for A) ChAdOx1 and B) BNT612b2, or with age at vaccination for C) ChAdOx1 and D) BNT612b2. E) Age at vaccination for each group (mean +/- SE). (DOCX) [file ppat.1010022.s007.docx]

**S4 Figure. Correlation between neutralising antibody titre and either days post-dose 1 or age at vaccination.**
