# Supplementary material for: Reduced neutralisation of the Delta (B.1.617.2) SARS-CoV-2 variant of concern following vaccination
Source: PLoS Pathog. 2021 Dec 2;17(12):e1010022. doi: 10.1371/journal.ppat.1010022 (PMC8639073; doi:10.1371/journal.ppat.1010022)
Supplement: Attachment — Submitted filename: Response to reviewer.docx [file ppat.1010022.s008.docx]

Reviewer #1: The manuscript “Reduced neutralization of the Delta (B.1.617.2) SARS-CoV-2 variant of concern

following vaccination” by C. Davis et al. compares the production of antibodies targeting S1 or RBD of SARS-CoV-2

and the neutralization efficacy of produced antibodies after vaccination with one or two doses of BNT162b2 and

ChAdOx1. They show that the BNT162b2 vaccine induces higher antibody production compared to ChAdOx1 after a

single dose as well as a double dose. Furthermore, they present reduced neutralizing activity against the mutant

viruses, B 1.351, B1.617.1, and B1.617.2 compared to Wuhan-Hu-1 virus after primary and secondary immunization

with BNT162b2 or ChAdOx1.

The paper is straight-forward and provides additional information on the effectiveness of vaccination against delta

mutations. However, the data is not optimally analyzed, the discussion overlong and repetitive and several references need to be introduced. These issues need to be addressed before it may be accepted.

Part II – Major Issues: Key Experiments Required for Acceptance

Please use this section to detail the key new experiments or modifications of existing experiments that should be

absolutely required to validate study conclusions.

Reviewer #1: 1) C. Liu also presented the reduced neutralization of the SARS-CoV-2 delta variant after Pfizer and

AstraZeneca vaccination. Although the results fit in general to the outcomes in this study, the significantly reduced

antibody neutralization ability shown in this study compared to Liu et al (2021), needs to be discussed.

Several studies have now addressed the neutralisation of emerging variants by sera from vaccinees and the majority concluded that there is a significant reduction in the sensitivity to neutralisation, particularly with B.1.351, but more recently with B.1.617. For example, Wang et al (Nature 2021) looked at the antibody resistance of B.1.351 and noted a reduction in sensitivity of 10.3-fold and 12.4-fold in sera from BNT162b2 (Pfizer)-vaccinated and mRNA-1273 (Moderna)-vaccinated individuals using a pseudotype-based assay. Cele et al (Nature 2021) used a live virus based microneutralisation assay and noted a reduction of 8 to 14-fold for B.1.351. In our study, we measured the reduction in neutralisation sensitivity of B.1.351 at 9.56-fold and B.1.617.2 at 11.30-fold for sera from BNT162b2-vaccinated individuals. Although the Wang and Cele studies preceded the emergence of B.1.617.2, there is an excellent concordance between the reduction we observed with B.1.351 and the studies of Wang et al and Cele et al., hence we can be confident that our estimate of the reduction in neutralisation of B.1.617.2 is likely to be consistent with previous experimental approaches.

The study cited by the reviewer, Liu et al 2021, obtained lower estimates of the reduction in neutralisation sensitivity (2.5 and 7.5 fold respectively for B.1.617.2 and B.1.351). Liu et al used sera collected from individuals following an accelerated vaccine regimen, with sera collected at 4-14 days post-two doses of BNT16b2 vaccine 3 weeks apart. Further, the reductions in Liu et al were calculated relative to a Vero cell-grown “Victoria” isolate of SARS-COV-2 which appears to be equivalent to Wuhan-hu-1 + S247R, and B.1.617.2 bearing A222V in a focus reduction neutralisation test (FRNT) format. While there are many variables that may have contributed to the estimates in Liu et al, it is likely that the shortened vaccination schedule coupled with the S247R and A222V-containing reference strains may have diminished the magnitude of the reductions noted by ourselves, Wang et al. and Celes et al. A full comparison of our data and the data of Liu et al has been added to the Discussion.

2) A correlation of RBD ELISA data to the in vitro neutralization values with the different pseudotypes would be of

value to the readers. It would allow to identify if the loss of neutralization is observed among people with strong

antibody responses, or across the board. Likewise, xy scatterplots of Wuhan versus variant(s) neutralization titers

would provide more information out of the existing dataset.

Figures to this effect have been added as supplementary materials

4) The discussion is in general overlong and repeats information from the results section. The discussion needs to be

shortened, and include references to similar studies (C. Liu et al.), or those that have been published in the meantime

since submission. The authors need to discuss the similarities and differences in the phenomena observed in this and other studies. It would be of particular interest to discuss the use of HIV pseudotypes versus VSV ones or of the

native SARS CoV-2 in neutralization assays. The existence of other studies should not preclude the publication of this

one, but it needs to be contextualized in light of the already available evidence.

The discussion has been edited to remove unnecessary repetition from the results and added discussion of Liu et al. included.

Part III – Minor Issues: Editorial and Data Presentation Modifications

Please use this section for editorial suggestions as well as relatively minor modifications of existing data that would

enhance clarity.

Reviewer #1: 1) The authors claim that the weaker antibody response of ChAdOx1 compared to BNT162b2 is due to

the difference in the age of participants. This claim would be more convincing if the authors would provide the

correlation of antibody titers and age. Furthermore, they should cite in this context a paper showing that antibody producing ability after administration of Pfizer and AstraZeneca vaccines decreases with age.

In the paper we do not “claim” that the weaker response in ChAdOx1 compared to BNT162b2 is due to differences in age, rather we note that there were disparate mean ages in the two vaccine groups and that this may have had a bearing on inter-group comparisons (based on previous studies by Wall et al and Bates et al.), although we did not observe a statistical difference in titre that correlated with age.
